# Relation of Decreased Functional Connectivity Between Left Thalamus and Left Inferior Frontal Gyrus to Emotion Changes Following Acute Sleep Deprivation

**DOI:** 10.3389/fneur.2021.642411

**Published:** 2021-02-26

**Authors:** Bo-zhi Li, Ya Cao, Ying Zhang, Yang Chen, Yu-hong Gao, Jia-xi Peng, Yong-cong Shao, Xi Zhang

**Affiliations:** ^1^Department of Neurology, Secondary Medical Center, National Clinical Research Center for Geriatric Disease, Chinese PLA General Hospital, Beijing, China; ^2^Department of Neurology, First Medical Center, Chinese PLA General Hospital, Beijing, China; ^3^Department of Medical Psychology, Eighth Medical Center, Chinese PLA General Hospital, Beijing, China; ^4^School of Biological Science and Medical Engineering, Beihang University, Beijing, China; ^5^Department of Psychology, Beijing Sport University, Beijing, China

**Keywords:** mood, functional connectivity, resting-state functional magnetic resonance imaging, thalamus, inferior frontal gyrus, acute sleep deprivation

## Abstract

**Objective:** The thalamus is a key node for sleep-wake pathway gate switching during acute sleep deprivation (ASD), and studies have shown that it plays a certain role in emotion changes. However, there are no studies on the association between the thalamus and emotion changes in ASD. In this study, we used resting-state functional magnetic resonance imaging (R-fMRI) to explore whether changes in the functional connections between the thalamus and other brain regions are related to emotion changes and further explored the function of the thalamus under total ASD conditions.

**Method:** Thirty healthy, right-handed adult men underwent emotional assessment according to the Profile of Mood States Scale and R-fMRI scans before and after ASD. The correlations between changes in functional connectivity between the thalamus and other brain regions and emotion changes were then studied.

**Results:** Positive emotions and psychomotor performance were reduced, and negative emotions were increased following ASD. The functional connections between the left thalamus and left middle temporal gyrus, left inferior frontal gyrus, right thalamus, right inferior temporal gyrus, left middle temporal pole gyrus, right calcarine, left cuneus, left rectus and left medial superior frontal gyrus were significantly altered. Decreased functional connectivity between left thalamus and left inferior frontal gyrus related to emotion changes following ASD.

**Conclusion:** This study finds that functional changes in the thalamus are associated with emotion changes during ASD, suggesting that the left thalamus probably plays an essential role in emotion changes under ASD conditions.

## Introduction

“Sleep deprivation” can be summarized as less sleep than is usually required. It can be acute or chronic, with the effects of a small amount of sleep deprivation accumulating over days, weeks or longer (1). With the rapid development of social modernization, people often actively or passively suffer from the adverse effects of chronic sleep deprivation (CSD) and acute sleep deprivation (ASD) (2, 3). Sleep deprivation leads to a series of neurological and behavioral changes that can significantly interfere with the brain's cognitive and emotional abilities. The short-term consequences of sleep deprivation include increased stress response, physical pain, decreased quality of life, emotional disorders and performance impairment (4), while the long-term consequences of sleep deprivation in healthy individuals include cardiovascular disease, weight-related problems, metabolic syndrome, dyslipidemia, and colorectal cancer (4). There is also an increase in all-cause mortality among men suffering from sleep deprivation (4). Furthermore, following sleep deprivation, brain function is significantly impaired in terms of attention, decision-making, spatial navigation, working memory and emotional and social processing (5, 6).

It is well known that sleep plays a critical role in emotional processing and regulation (5). Functional magnetic resonance imaging (fMRI) studies have shown that sleep-deprived people have altered emotional brain networks, mainly in the limbic system (5). Compared with healthy people, the volume, activity and functional connections of the amygdala, insula, cingulate area and prefrontal lobe in patients with emotional disorders, such as various types of anxiety and bipolar disorder, are significantly changed, which further confirms that these are the main brain areas responsible for the related emotions (7–9). Correlations between these brain regions and emotion have mainly been identified in studies of people with CSD, but a growing number of studies show that ASD has a wide range of effects on emotion (10, 11). Further research shows that the amygdala, anterior insula, medial prefrontal cortex and anterior cingulate cortex are also significantly altered under ASD, and associated with emotion changes caused by ASD (6, 12). The above research findings provide preliminary evidence that the areas of the brain associated with emotion changes are largely the same in both ASD and CSD.

All brain regions are affected by emotions (13). However, the research on human brain by fMRI mainly focuses on the higher cortical regions, and there are few studies on the involvement of subcortical structures in emotional changes. As an important structure involved in the sleep-wake pathway, the thalamus has been shown to be involved in alert-related brain cognitive functions (6). And in certain chronic progressive diseases such as anxiety disorder and insomnia, the thalamus is involved in the emotional neural networks. Research into the emotional dysregulation circuit shows that the thalamus plays a particular role in emotion changes (14, 15). Furthermore, studies have shown that CSD (insomnia, etc.) can cause functional changes in the thalamic-emotional core region (16, 17). To date, the emotional role of the chronically sleep-deprived thalamus has been documented, but the fact that the thalamus is involved in emotion changes has not been adequately studied. At the same time, there were almost no studies have investigated the emotional role of the thalamus during ASD. While the vast majority of studies show that the thalamus plays a key central role in the sleep-wake pathway and involved in a variety of brain cognitive functions (6, 18–21), and the thalamus is located anatomically in the core area of the brain, Therefore, there is good evidence to speculate that the thalamus may play a unique role in emotion changes caused by ASD. However, ASD and insomnia show extensive changes in the microstructure of gray matter and share common but different neurobiological characteristics in brain morphology (22). It is necessary to provide direct evidence to test this hypothesis.

We hypothesized that the functional connections between the thalamus and brain regions proven to be associated with emotion would be altered following ASD, and the functional changes between the thalamus and other regions would correlate with the emotion changes. To test this hypothesis, we devised a within-subject statistical design of 36 h total ASD, then used f-MRI to assess whether changes in the functional connections between the thalamus and other brain regions were correlated with emotion changes under ASD conditions.

## Materials and Methods

### Participants

We recruited 30 young male college students (right-handed, age range: 20–30 years) and offered them a financial reward to participate in this study. During the recruitment process, we explained in detail the main purpose of the study and its whole process, as well as the possible risks and countermeasures. All subjects voluntarily signed their informed consent. Prior to the beginning of the experiment, we invited qualified Chinese specialists to conduct standardized physical examinations. The main forms of medical examination were subjective and objective physical examinations, as well as related self-reporting scale tests, to exclude subjects who may have serious diseases or be at risk of accidents during the experiment. The inclusion criteria we set were as follows: ① No disease history of the circulatory system or respiratory system, no nervous system structure or function impairment, and no history of severe infectious disease, mental disorder or sleep disorder; ② No colds or other diseases in progress that may affect the experiment; ③ The subjects had regular daily life and rest habits, and their score was <7 on the Pittsburgh Sleep Quality Index (PSQI) scale; ④ No significant events leading to the possible emotional fluctuation of the subjects had occurred within 1 month before the experiment; ⑤ Subjects were required to carry out their daily activities in accordance with the standard procedures in the week before the start of the experiment, and advised to refrain from consuming stimulating beverages, carbonated beverages, tea, coffee and certain foods, as well as refraining from smoking. The study was approved by the Research Ethics Committee of Beihang University (Beijing, China). We conducted it in strict accordance with the protocol approved by the Ethics Committee, and strictly followed the requirements of the Hellenic Declaration.

### Behavioral Measures

In this study, we used the Profile of Mood States Scale (POMS) to assess emotion changes (23). The advantage of this scale is that it can provide a reasonable and timely assessment of emotion changes through changes in the score. It consists of a questionnaire containing 65 items over six sections: anxiety, anger, fatigue, depression, confusion, and vitality. The scores for these items range from 0 (not at all) to 4 (very). According to the scoring principle, the scores for the 65 items are added to obtain the scores for the six sections. The top five sections form the negative emotional state assessment, and the sixth section is the positive emotional state assessment. The total score is the sum of the top five sections minus the score for the sixth section. The higher the negative emotion score, the more serious the emotional disorder, while the higher the positive score, the better the emotional state. In our study, Cronbach's alpha coefficients were satisfactory (Cronbach's alpha is 0.909).

We used the Chinese version of the PSQI, which has good psychometric properties (24). The PSQI is a self-reporting questionnaire which measures sleep quality over the previous month (25). Scores for each sleep item range from 0 (not at all) to 3 (maximum dysfunction). The scores for the sleep items are then added according to the coefficient to obtain the overall sleep quality score. We classified PSQI scores of equal to or >7 as low sleep quality.

### Procedures

**Figure 1:**
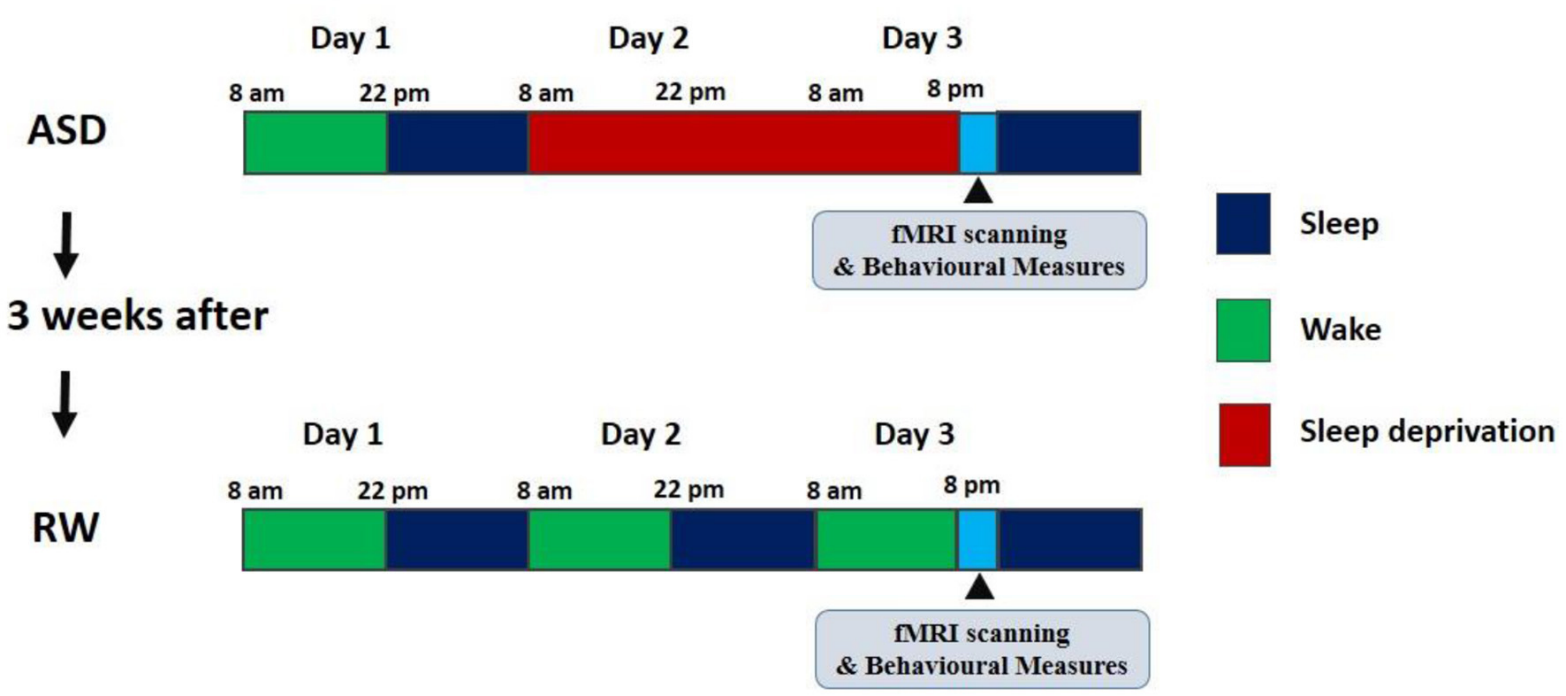
The design of the experiment. ASD, acute sleep deprivation; RW, rested wakefulness.

The design of the experiment is presented in Figure 1.

We experimented in the sleep laboratory of Beihang University and the magnetic resonance room of the General Hospital of the People's Liberation Army of China. All subjects participating in the experiment were divided into four batches to monitor their status more accurately and reduce the bias caused by an excessive number of subjects during the scale assessment and R-fMRI scan. During the experiment, we ensured that there were two experimenters (no fewer than one operator with a medical qualification) to guide the subjects to complete the relevant experiment contents and supervise their physical health status and experiment cooperation degree.

After preparing for the experiment in their living quarters, the subjects arrived at the laboratory at 8:00 on the first day of the experiment. They wore exercise watches to record their activity and sleep during the experiment. In the daytime of Day 1, they familiarized themselves with the experimental site and completed the evaluation. From 22:00 on Day 1 to 8:00 on Day 2, the subjects slept for at least 8 h under the supervision of the operator. After these preparations were completed, ASD started at 8:00 on Day 2 and ended at 20:00 on Day 3, during which time all subjects completed 36 h of continuous sleep deprivation. During the experiment, subjects were allowed to carry out daily activities, including playing games, reading, sitting, eating, and chatting. During the whole experiment stage, especially during the sleep deprivation stage, at least two experimenters were on hand at the same time to monitor the status of the subjects, thereby avoiding the occurrence of influencing factors such as naps during the sleep deprivation period.

We performed two R-fMRI scans during rested wakefulness (RW), and they were carried out at least 3 weeks apart to dull the effects of the exercise. The R-fMRI scan during RW used the same scan sequence as the R-fMRI scan during ASD and was performed within the same period, while the subjects completed their POMS evaluation immediately before each scan. The two scans were performed by a 3T Siemens MAGNETOM Skyra (Siemens Medical Solutions, Germany) located in the General Hospital of the People's Liberation Army. During each scan, the T1 sequence was scanned first to obtain high resolution T1-weighted anatomical images (176 images). Next, R-fMRI data collection was performed for 8 min (240 images per time). At the beginning of the scan, the subjects were asked to lie on their backs on an MRI bed with their heads fixed in a sponge and bandage. During the scan, the subjects were told to close their eyes, think of nothing and try to keep their heads and bodies still. To ensure that the subjects were awake during the scan, the operator communicated with them through a microphone before each scan to remind them to stay awake, and they were again asked if they were awake between scans of different sequences. Throughout each scan, the operator monitored the subject's body movements and other states through the viewing window. After each scan, the subjects were asked if they had been awake during the scan.

### Data Processing

We used CONN toolbox software (Version 18a, Neuroimaging Informatics Tools and Resources Clearinghouse; http://www.nitrc.org/projects/conn) and SPM software (Version 12, University College London, http://www.fil.ion.ucl.ac.uk/spm) to preprocess the R-fMRI images. The two types of software are both cross-platform software based on MATLAB (Version R2018a, MathWorks, Inc. United States). First, the T1-weighted images underwent translation, segmentation and MNI normalization. Second, the first 10 volumes of the functional images were disregarded. Third, functional slice-timing correction and subject motion estimation and correction were carried out. We excluded all subjects whose mean head movements were more than 2 mm and 2°. While in the remaining subjects, the images were deleted if the head moved more than 0.5° or 0.5 mm from the adjacent images. Finally, functional indirect segmentation and normalization were carried out. In the space standardization step, the functional images were indirectly normalized to the standard space through the corresponding structural images, and the normalized bias correction is generated. The EPI template was used to normalize the structural images directly into the standard Montreal Neurological Institute (MNI) 152 standard space (voxel size of 3 × 3 × 3 mm). The full width at half maximum in smoothing was 6 mm, and the low frequency filter was 0.01–0.08 Hz. Fisher's transformation was used to normalize the distribution of variables (26, 27).

#### Functional Connectivity Analysis

A measure of functional connectivity between each pair of seed regions was typically calculated by region of interest to the region of interest (ROI-to-ROI) analysis. The seed regions to be studied were selected using the CONN toolbox's Automated Anatomical Labeling (AAL), and the location of these seed points was based on the details given by Tzourio-Mazoyer (28). A total of 90 seed regions were selected through AAL (18 seeds were removed from the cerebellar region). We then calculated the functional connectivity of the left and right thalami to the other seed regions separately for each subject. Next, Pearson correlation analysis was performed on the time series of each seed region. Finally, the Fisher Z-transformation was used to transform the correlation coefficient of each voxel and smooth the obtained data. After several corrections to the dataset, the inter-condition effect was considered to be significant at *p* < 0.05, and the group-level false discovery rate (FDR) correction *p-*value was <0.05.

#### Behavioral Measures and Correlation Analysis

SPSS (Version 21.0, IBM, Inc., USA) software was used to process the collected POMS data and other demographic data. First, descriptive statistics were carried out on each index, and a normality test was carried out on each item result and total POMS score. The measurement data was presented in the form of mean ± standard deviation. Later, a paired sample *t*-test was used for each section score and total POMS score before and after ASD, and *p* < 0.05 was considered statistically significant. Finally, the changes in functional connectivity coefficient between the thalamus and various brain regions (*p* < 0.05, FDR-corrected) were compared with the difference between POMS scores before and after ASD. Under the premise that the *p*-value was < 0.05, we defined the correlation coefficients (*r*) ≤ 0.4 as low correlation, 0.4 < *r* ≤ 0.6 as moderate correlation, and 0.6 < *r* as high correlation.

## Results

### Initial Data Quality Assessment

In the demographic description, we gave a statistical description of 28 subjects with complete data. They were all males with an average age of 24.48 ± 2.57 years, average height of 175.93 ± 5.01 cm, and average Body Mass Index (BMI) of 23.64 ± 1.73. Their PSQI scores were all <7, and their average score was 3.37 ± 1.19.

### Behavioral Measures and Correlation Analysis

We used POMS to assess emotion changes before and after ASD. These scores were in line with the normal distribution, and a paired sample *t*-test was used to measure emotion changes. Following ASD, there were statistically significant increases in scores for anxiety (*t* = 2.635, *p* = 0.014), anger (*t* = 2.066, *p* = 0.049), fatigue (*t* = 5.217, *p* < 0.001) and confusion (*t* = 4.719, *p* < 0.001), and there was a statistically significant decrease in scores for vitality (*t* = −6.464, *p* < 0.001). The total POMS score also showed a statistically significant increase (*t* = 6.215, *p* < 0.001). In addition, decrease in depression was not statistically significant (Table 1).

**Table 1:** POMS statistics: comparisons between RW and ASD (paired *t*-test, *n* = 28).

**Mood**	**RW**	**ASD**	**Mean (ASD > RW)**	**SD**	***t***	**Sig. (2 tailed)**
Confusion	13.04 ± 2.40	15.00 ± 2.85	1.96	2.20	4.72	<0.001
Anxiety	14.79 ± 3.67	16.29 ± 3.73	1.50	3.01	2.64	0.014
Depression	20.61 ± 6.37	22.21 ± 7.29	1.61	5.21	1.63	0.114
Anger	16.54 ± 5.39	18.32 ± 6.63	1.79	4.57	2.07	0.049
Vitality	27.57 ± 4.39	21.04 ± 7.17	−6.54	5.35	−6.46	<0.001
Fatigue	11.14 ± 3.14	15.82 ± 5.21	4.68	4.75	5.22	<0.001
Total score	48.54 ± 20.07	66.61 ± 25.44	18.07	15.39	6.22	<0.001

**Figure 2:**
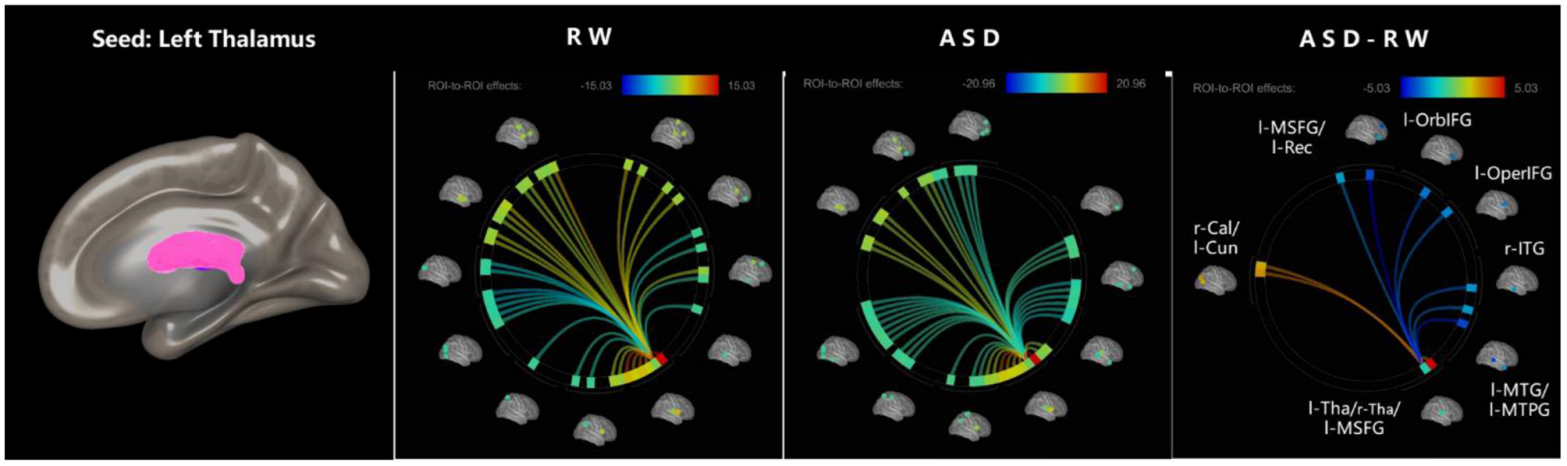
ROI-to-ROI functional connectivity of left thalamus during RW, ASD, and ASD > RW conditions. False discovery rate-corrected (*p* < 0.05) for ROI-to-ROI tests. The functional connectivity between l-Tha and l-OperIFG, l-OrbIFG, l-MTPG, l-Rec, l-MTG, and r-ITG decreased during ASD. The functional connectivity between l-Tha and l-Cun, r-Cal, l-MSFG, and r-Tha increased during ASD. l-MSFG, left medial superior frontal gyrus; l-MTPG, left middle temporal pole gyrus; l-OrbIFG, left orbital inferior frontal gyrus; l-OperIFG, left opercular inferior frontal gyrus; l-Rec, left rectus; l-MTG, left middle temporal gyrus; r-ITG, right inferior temporal gyrus; r-Tha, right thalamus; l-Cun, left cuneus; r-Cal, right calcarine; ROI, region of interest; ASD, acute sleep deprivation; RW, rested wakefulness.

**Figure 3:**
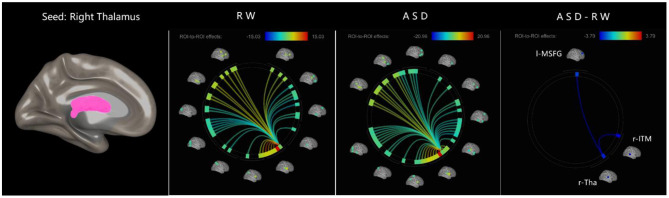
ROI-to-ROI functional connectivity of left thalamus during RW, ASD, and ASD > RW conditions. False discovery rate-corrected (*p* < 0.05) for ROI-to-ROI tests. The functional connectivity between r-Tha and r-ITM, l-MSFG decreased decreased during ASD. r-Tha, right thalamus; r-ITM, right middle temporal gyrus; l-MSFG, left medial superior frontal gyrus; ROI, region of interest; ASD, acute sleep deprivation; RW, rested wakefulness.

The effect sizes of functional connectivity between the thalamus and whole-brain ROIs significantly changed (*p* < 0.05, FDR-corrected) under ASD > RW conditions are shown in Table 2. The ROI-to-ROI analysis demonstrated a decreased functional connectivity between the thalamus and other brain regions mainly distributed in the frontal temporal lobe, including the left middle temporal gyrus (l-MTG), right middle temporal gyrus (r-MTG), left middle temporal pole gyrus (l-MTPG), right inferior temporal gyrus (r-ITG), left orbital inferior frontal gyrus (l-OrbIFG) and left opercular inferior frontal gyrus (l-OperIFG). In contrast, an increase in functional connectivity between the left thalamus (l-Tha), left medial superior frontal gyrus (l-MSFG), right thalamus (r-Tha), left cuneus (l-Cun) and right calcarine (r-Cal) occurred during ASD. Figure 2 shows the ROI-to-ROI functional connectivity of the left thalamus under RW, ASD and ASD > RW conditions. Figure 3 shows the ROI-to-ROI functional connectivity of the r-Tha under the three sets of conditions.

**Table 2:** ROI-to-ROI functional connectivity statistics: comparisons between RW and ASD scans (ASD > RW, paired *t*-test, *n* = 28).

**Target region**	**Abbreviation**	**AAL label**	**MNI center**	***t***	**Uncorrected *p*-value**	**FDR-corrected *p*-value**
**Left thalamus**	**l-Tha**	**Thalamus_L**	–**12**, –**18, 8**			
Left medial superior frontal gyrus	l-MSFG	Frontal_Sup_Med_L	−6, 49, 31	5.03	<0.001	0.003
Left middle temporal gyrus	l-MTG	Temporal_Mid_L	−57, −34, 30	−4.41	<0.001	0.007
Left orbital inferior frontal gyrus	l-OrbIFG	Frontal_Inf_Orb_L	−37, 31, −12	−3.81	<0.001	0.022
Right thalamus	r-Tha	Thalamus_R	12, −18, 8	3.35	0.002	0.045
Right inferior temporal gyrus	r-ITG	Temporal_Inf_R	53, −31, −22	−3.3	0.003	0.045
Left cuneus	l-Cun	Cuneus_L	−7, −80, 27	3.26	0.003	0.045
Left opercular inferior frontal gyrus	l-OperIFG	Frontal_Inf_Oper_L	−49, 13, 19	−3.19	0.004	0.045
Left middle temporal pole gyrus	l-MTPG	Temporal_Pole_Mid_L	−37, 15, −34	−3.12	0.004	0.047
Right calcarine	r-Cal	Calcarine_R	15, −73, 9	3.04	0.005	0.049
Left rectus	l-Rec	Rectus_L	−6, 37, −18	−3.01	0.006	0.049
**Right thalamus**	**r-Tha**	**Thalamus_R**	**12**, –**18, 8**			
Right middle temporal gyrus	r-MTG	Temporal_Mid_R	56, −37, −1	−3.79	0.001	0.049
Left medial superior frontal gyrus	l-MSFG	Frontal_Sup_Med_L	−6, 49, 31	−3.66	0.001	0.049

*ROI, region of interest; FDR, false discovery rate; ASD, Acute sleep deprivation; RW, rested wakefulness; AAL, Automated Anatomical Labeling; MNI, Montreal Neurological Institute*.

**Figure 4:**
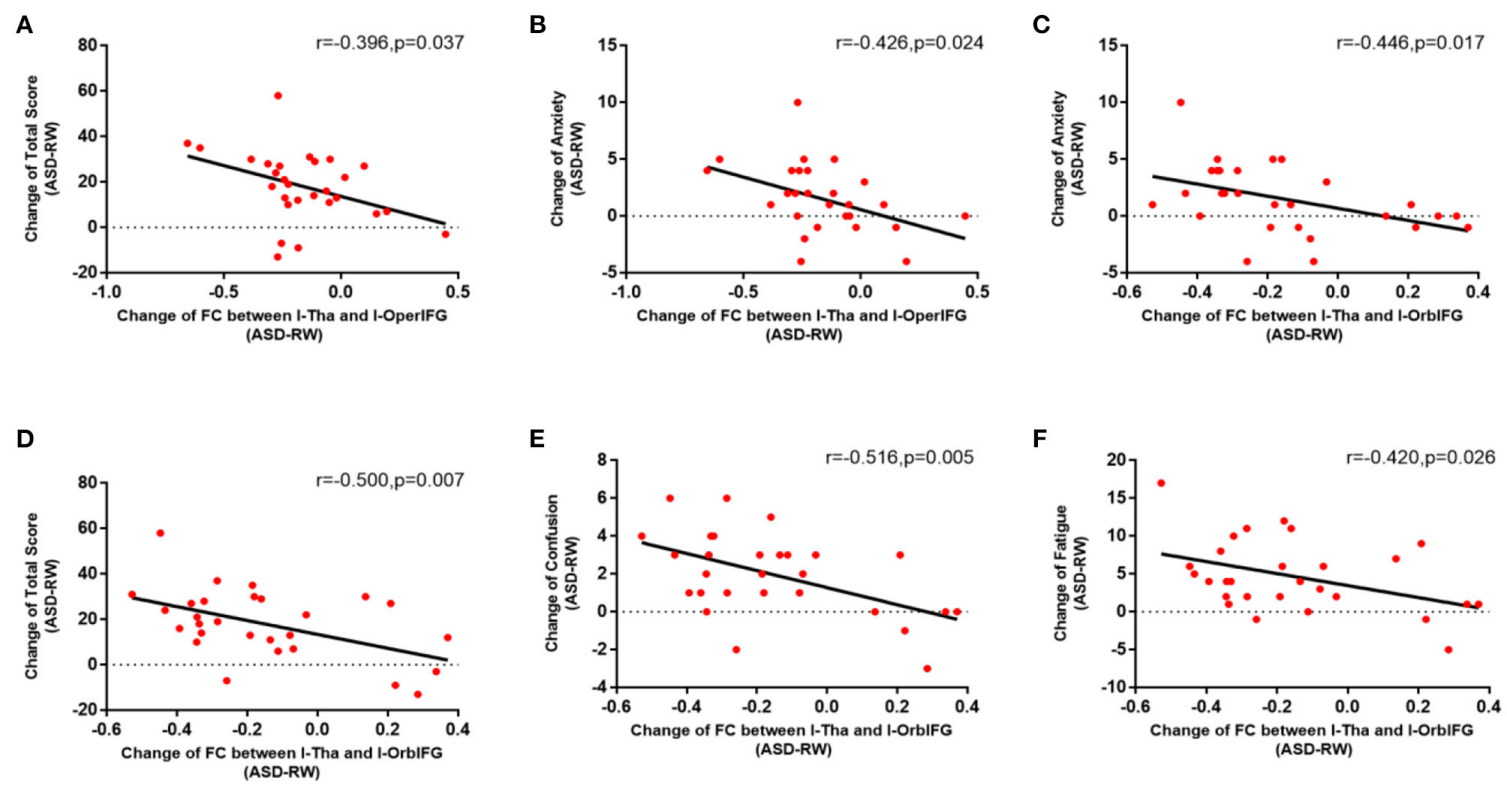
Relation of functional connectivity between left thalamus and other brain regions to emotion changes following acute sleep deprivation (ASD > RW, *n* = 28). The decrease in functional connectivity between l-Tha and l-OperIFG **(A)**, between l-Tha and l-OrbIFG **(D)** was significantly negatively correlated with the Change of total score of the POMS. The decrease in functional connectivity between l-Tha and l-OperIFG **(B)**, between l-Tha and l-OrbIFG **(C)** was significantly negatively correlated with the change of anxiety. The decrease in functional connectivity between l-Tha and l-OrbIFG **(E,F)** was significantly negatively correlated with the change of confusion and fatigue. FC, functional connectivity; ASD, acute sleep deprivation; RW, rested wakefulness; l-Tha, left thalamus; l-OperIFG, left opercular inferior frontal gyrus; l-OrbIFG, left orbital inferior frontal gyrus.

The correlation analyses showed that emotion changes are associated with changes in the functional connections between the thalamus and parts of brain regions following ASD (Figure 4). The decrease in functional connectivity between the left thalamus and left orbital inferior frontal gyrus was correlated with change in emotion: *r* (anxiety) = −0.446, *p* = 0.017; *r* (confusion) = −0.516, *p* = 0.005; *r* (fatigue) = −0.420, *p* = 0.026; *r* (total score) = −0.500, *p* = 0.007. The decrease in functional connectivity between the left thalamus and left opercular inferior frontal gyrus was also correlated with emotion changes: *r* (anxiety) = −0.426, *p* = 0.024; *r* (total score) = −0.396, *p* = 0.037. However, no extra significant correlation was found between the alterations of functional connectivity and emotion changes during ASD (Table 3).

**Table 3:** Correlation between changes in functional connectivity and changes in POMS (*n* = 28).

**Mood**	**Brain regions (changed to the left thalamus)**									
	**l-MSFG**	**l-MTPG**	**l-OrbIFG**	**l-OperIFG**	**l-Rec**	**l-MTG**	**r-ITG**	**r-Tha**	**l-Cun**	**r-Cal**
Confusion	−0.117	0.041	−0.516^*^	−0.116	−0.158	0.022	0.031	0.181	0.335	0.109
Anxiety	−0.253	0.114	−0.446^*^	−0.426^*^	−0.123	−0.277	−0.26	0.278	0.368	0.052
Depression	−0.115	−0.096	−0.199	−0.315	−0.097	−0.252	−0.029	0.292	0.385^*^	0.064
Anger	−0.114	0.161	−0.172	−0.242	0.12	−0.206	0.039	0.271	0.114	0.017
Vitality	0.285	−0.07	0.261	0.15	0.245	0.009	−0.067	−0.137	0.208	−0.147
Fatigue	−0.176	−0.163	−0.420^*^	−0.21	−0.257	−0.249	−0.143	0.159	0.192	0.207
Total Score	−0.292	0.018	−0.500^*^	−0.396^**^	−0.208	−0.277	−0.066	0.356	0.271	0.167

Pearson correlation analysis:

* *p < 0.05 and 0.4 < r ≤ 0.6, moderate correlation*;

** *p < 0.05 and r ≤ 0.4, low correlation*.

*l-MSFG, left medial superior frontal gyrus; l-MTPG, left middle temporal pole gyrus; l-OrbIFG, left orbital inferior frontal gyrus; l-OperIFG, left opercular inferior frontal gyrus; l-Rec, left rectus; l-MTG, left middle temporal gyrus; r-ITG, right inferior temporal gyrus; r-Tha, right thalamus; l-Cun, left cuneus; r-Cal, right calcarine*.

## Discussion

The role played by the thalamus in emotion changes following ASD remains unclear, although ASD can lead to emotion changes (6, 12), and the thalamus has been shown to be involved in emotion changes under CSD conditions. As an essential node in the sleep pathway, the role of the thalamus in sleep is beyond doubt (6). It is of great interest to explore the role of the thalamus in emotion changes following ASD. Our study provides preliminary evidence that the thalamus as a node is associated with emotion changes. In this study, the results of POMS confirmed that negative emotions significantly increased and positive emotions significantly decreased before and after ASD. Next, through R-fMRI analysis, significant changes were found in the functional connections between the thalamus and the brain regions that are mainly responsible for emotional processing. Finally, through correlation analysis, significant changes in the functional connections between the thalamus and related brain regions were found to be closely related to emotion changes.

### Emotion Changes Following ASD

Compared to the RW state, there were significant emotion changes following ASD. The total score of the POMS scale and its five parts of anxiety, confusion, anger, vitality and fatigue all changed significantly. This is consistent with other relevant studies (10, 11, 29–31). Furthermore, these results largely support those of Short and Louca (32) and Babson et al. (31), which show that ASD is sensitive to different emotional deficits under different emotional states, and has a more significant impact on confusion, energy and fatigue than on depression, anxiety and anger (31, 32). Moderate sleep deprivation aggravates confusion, vigor and fatigue, and emotional states such as anger and anxiety often worsen with more severe sleep deprivation, especially after complete sleep deprivation (31, 32). We agree with Mischel's reasoning about this changing trend and argue that people with different emotional states are sensitive to sleep deprivation differently. It's important to note that our study found no significant change in depressive mood following ASD. This is consistent with relevant studies to a certain extent, as ASD is an effective treatment for depression, with well-documented efficacy around 50% (33, 34). This may explain the absence of significant changes in depression in healthy subjects following ASD. Besides, our subjects within each group knew each other and did not undergo high-intensity experimental content, which may have reduced the degree of specific negative emotion changes such as depression during the experiment.

### Changes in Brain Functional Connectivity

The functional connectivity between the thalamus and other brain regions was significantly changed following ASD, and these regions are mainly located in the frontal and parietal cortex which are involved in almost all functions related to emotion (5–7, 35). We observed significantly changed functional connections between the thalamus and the left inferior frontal gyrus, left middle temporal gyrus, right middle temporal gyrus, right inferior temporal gyrus and left medial superior frontal gyrus, all of which are involved in emotional function to some extent. Actually, almost the entire brain network is involved in emotional functions (36), and sleep deprivation can reliably trigger changes in negative emotional processes including irritability, anxiety, aggression, and mood swings (6). Meanwhile, although the thalamus is an essential node of wakefulness switching in the sleep-wake pathway, we noticed no significant change in the functional connections between the thalamus and the amygdala, an emotion-processing region of the limbic system which is susceptible to ASD (6, 37). These changes in functional connections between the thalamus and other brain regions associated with emotion changes strongly suggest that the thalamus may be involved in emotion changes to some extent. This may support the idea that in emotional experience and the perception of a set of discrete categories of emotion, a group of interactive brain regions is usually involved in emotional and non-emotional basic psychological operations (37).

### Relation of Altered Functional Connectivity to Emotion Changes

Our study of the relation of altered functional connectivity to emotion changes further confirms that the thalamus is involved in emotion changes following ASD. It was found that the functional connections between the left thalamus and left orbital inferior frontal gyrus were negatively correlated with the total POMS score and confusion, anxiety and fatigue. The orbital frontal gyrus consists of medial ventral parts of the superior, middle and inferior frontal gyrus. It processes emotional responses to internal cues and modulates emotions and rewards in the decision-making process. In particular, the left orbital inferior frontal gyrus is closely related to emotion changes, and the impairment of the orbital frontal regulation of limbic emotional processing is considered the cause of the bipolar disorder (38, 39).

Our study also observed that the functional connections between the left thalamus and left opercular inferior frontal gyrus were negatively correlated with the total POMS score and anxiety (Table 3). The left opercular inferior frontal gyrus is an essential area for language processing (40), and the relatively few relevant studies have pointed out that there is a specific correlation between the left opercular inferior frontal gyrus and social anxiety disorder (15, 41–43).

The above results show that the functional connections between the left thalamus and left inferior frontal gyrus are correlated with emotion changes following ASD, especially anxiety (Figure 4). The inferior frontal gyrus is comprised of the orbital inferior frontal gyrus, triangular inferior frontal gyrus and opercular inferior frontal gyrus (44). The study found significant changes in frontal lobe function during sleep deprivation (45), and certain studies have suggested that functional changes in the left inferior frontal gyrus during sleep deprivation are associated with anxiety, depression and mood swings (7, 43, 46, 47). Given the various functions of the left inferior frontal gyrus, it is difficult to distinguish whether it is the responsible center or relay station of emotion changes according to the existing research, but it is clear that the left inferior frontal gyrus is an important hub of the emotional pathways.

The left thalamus engages in a variety of emotion changes presented in many diseases such as anxiety disorder, bipolar disorder, posttraumatic stress disorder, major depressive disorder, and hyperalexithymia (8, 48). Patients with high stress are specifically associated with lesions in the left thalamus (49) since both the left thalamus and left inferior frontal gyrus are involved in a variety of emotion changes, and the inferior frontal gyrus interacts closely with the thalamic nuclei (44). Furthermore, studies have suggested that CSD is characterized by structural and functional changes between the thalamus and left inferior frontal gyrus (50–52). Meanwhile, the functional connections between the thalamus and frontal lobe seem sensitive to mild sleep-wake changes (53) which are similar to those observed following ASD in our study. Most importantly, our experiment shows that the functional connections between the left thalamus and left inferior frontal gyrus are strongly associated with emotion changes such as anxiety. Taken together, there is sufficient preliminary evidence to demonstrate the involvement of the thalamus in emotion changes induced by ASD.

Furthermore, although the functional connections between the bilateral thalamus and medial superior frontal gyrus decreased, there was no clear correlation between this decrease and emotion changes. The medial superior frontal gyrus, one of the most important brain regions in the emotional network, showed altered functional connections with the bilateral thalamus during ASD, but this did not seem to be the leading cause of the emotion changes. Meanwhile, our results showed that the functional connections between the thalamus and other brain regions mainly responsible for emotion were not significantly changed following ASD, which further suggests that the brain regions affected by ASD and those affected by CSD may not be entirely consistent.

We further speculate that the emotion changes under ASD related to the left inferior frontal gyrus and thalamus are not entirely consistent with the traditional emotional network. The thalamus has been observed to participate in a variety of functional networks that have different response patterns (54). This suggests that functional tissues allow spatially overlapping networks of resting states of the brain, facilitating the description of various interpersonal relationships between overlapping regions and different functional systems in other regions of the brain (54). This partly explains the decreased functional connections between the left thalamus and left inferior frontal gyrus associated with emotion changes after sleep deprivation. It is also possible that there is a network mechanism for new emotion changes in the context of ASD.

In summary, our study finds that functional changes in the thalamus following 36 h of total ASD are associated with emotion changes. The changes in functional connection between the left thalamus and left inferior frontal gyrus were negatively correlated with emotion changes, and the thalamic-related emotion regulation circuit was affected. This means that the left thalamus plays a vital role in emotion changes following ASD.

## Limitations of the Study

First, the subjects of our study were all young male men limited to the experimental conditions, and literature has pointed out that gender may have different effects on emotional state (e.g., depression) during sleep deprivation (55). Gender and age may affect the accuracy of the experimental conclusion, we should take the gender differences into consideration in future fMRI studies, especially the treatment of brain-related diseases. We will build on the present study and refine our study in future studies to cover left-handedness and sleep deprivation in women. Second, the thalamus is a big ROI in AAL, and calculations based on this seed point may be general. However, for the selection of ROI in AAL, we mainly considered that this template was widely used, especially in the previous thalamic research articles. Therefore, we chose this conservative seed point in this exploratory study. Furthermore, we will study the function of the thalamus by using a more detailed method in further studies to further improve this conservative but robust conclusion.

## Data Availability Statement

The raw data supporting the conclusions of this article will be made available by the authors, without undue reservation.

## Ethics Statement

The studies involving human participants were reviewed and approved by Research Ethics Committee of Beihang University (Beijing, China; BM20180040). The patients/participants provided their written informed consent to participate in this study.

## Author Contributions

B-zL contributed to performing the experiments, processing the data and drafting the paper. YCa contributed to processing the data and drafting the paper. YZ and YCh contributed to performing the experiments and acquiring the data. Y-cS, Y-hG, and XZ are the guarantors of the study. All authors made substantial contributions to this work and approved it for publication.

## Conflict of Interest

The authors declare that the research was conducted in the absence of any commercial or financial relationships that could be construed as a potential conflict of interest.
